# CircRNF111 Contributes to Adipocyte Differentiation by Elevating *PPARγ* Expression via miR-27a-3p

**DOI:** 10.1080/15592294.2022.2145058

**Published:** 2022-11-15

**Authors:** Xuemei Shen, Jia Tang, Yongzhen Huang, Xianyong Lan, Chuzhao Lei, Hong Chen

**Affiliations:** a Engineering Research Center of Sichuan Province Higher School of Local Chicken Breeds Industrialization in Southern Sichuan, College of Life Science, Leshan Normal University, Leshan, China; b Key Laboratory of Animal Genetics, Breeding and Reproduction of Shaanxi Province, College of Animal Science and Technology, Northwest A&F University, Yangling, Shaanxi, China; c College of Animal Science, Xinjiang Agricultural University, Urumqi, China

**Keywords:** Bovine, adipocyte differentiation, circular RNAs, *pparγ*, miR-27a-3p

## Abstract

The content and distribution of adipocytes is an important factor that affects meat quality. Previous studies showed that circRNAs are involved in various physiological processes. Nevertheless, more research is needed to investigate the function of circRNAs in adipogenesis. The present study examines the effects of circRNF111 on adipogenesis of bovine preadipocyte and aims to elucidate the underlying molecular mechanisms. In our study, the sequence signature of circRNF111 was identified using bioinformatics, RNA-FISH, and sequencing. Mechanistically, knockdown or exogenous expression of circRNF111 in preadipocytes was done to prove the functional significance of circRNF111. Combined with bioinformatics, a dual fluorescein reporter system, and immunoprecipitation, the interaction between circRNF111, miR-27a-3p, and the target gene *PPARγ* was verified. The results reveal that circRNF111 is positively correlated with adipocyte differentiation. The newly identified bovine circRNF111 functions as a miR-27a-3p sponge to rescue the inhibitory effect of miR-27a-3p on the *PPARγ* gene, thereby promoting adipogenesis.

## Introduction

In the beef industry, carcase quality and value are reflected in the protein and fat content. Adipocytes are the major contributors to the marbling component needed to satisfy consumer preferences. However, along with the deposition of intramuscular fat that composes marbling, a large amount of fat accumulates as subcutaneous fat and visceral fat, which is energetically unfavourable and reduces the efficiency of production [1]. Therefore, it is necessary to study the mechanism of fat deposition. Adipogenesis is governed by a tightly controlled cascade of transcription factors coordinating hundreds of genes to establish the mature adipocyte phenotype [2]. Recent studies point out that adipogenesis is controlled by a network of multiple genes and non-coding RNAs (ncRNAs) with pleiotropic effects [3–5]. Therefore, analysing the associations of ncRNAs linked with economically important genes may be useful.

The introduction of new biotechnology and next-generation sequencing technology enabled the discovery of developmental genes. Several critical genes have been demonstrated to mediate adipogenesis, including peroxisome proliferative activated receptor gamma [*PPARγ*] [6], sterol regulatory element binding transcription factor 1 [*SREBP1*] [7], and CCAAT/enhancer-binding protein alpha [*C/EBPα*] [8]. *PPARγ* is a key molecule in adipogenesis. It induces fibroblasts or preadipocytes to differentiate into adipocytes and is abundantly expressed in adipose tissue [9,10]. It controls target genes in adipogenesis, lipid transport, and insulin sensitization [9,11] by directly or indirectly enhancing the transcription of genes-encoding proteins such as lipoprotein lipase (*LPL*), fatty acid binding protein (*FABPs*), and liver X receptor α [12–14].

Except for critical coding genes, numerous ncRNAs have also been shown to mediate adipogenesis [15]. For instance, microRNAs (miRNAs) are small, single-stranded, ncRNA molecules that impede protein production by interacting with the 3’untranslated region (3’UTR) of the target mRNAs [16]. miRNAs regulate the differentiation of adipocytes by inhibiting the expression of adipogenic-related genes. The miR-130b inhibits adipogenesis by inhibiting the transcription of *PPARγ* [17], while miR-15a/b could promote adipogenesis by targeting *Foxo*1 [18]. Additionally, miR-326 targets *C/EBPα* to inhibit fat production and proper differentiation of human adipose-derived stem cells [19]. Moreover, miR-378a-3p has been shown to be a direct transcriptional target of mitogen-activated protein kinase 1, which upregulates the genes required for fatty deposition [20]. In addition, Wu et al. reported that miR-27a-3p is a crucial regulator of human adipogenesis [21].

Circular RNAs (circRNAs) are another type of endogenous ncRNAs formed by reverse splicing whose covalently closed loop structure is relatively stable without 5’-cap and 3’-polyadenylated tail [22]. In recent years, circRNAs have been verified to mediate multiple physiological processes [23,24]. CircRNAs can act as competitive endogenous RNAs (ceRNAs), regulating the activity of miRNAs or directly modulating gene expression at both the transcription and splicing levels. Or they can function in cells by encoded protein [25,26]. Among them, circRNAs exert the function of regulating cell physiological processes by sponging miRNAs [22,27]. For example, circINSR inhibits preadipocyte adipogenesis by alleviating the inhibition of miR-15/16 against the target genes *FOXO1* and *EPT1* [28]. CircTshz2-1 and circArhgap5-2 are indispensable regulators of fat formation [5]. CircFUT10 could inhibited the differentiation of adipocytes by regulating let-7c/PPARGC1B signalling [29]. Several studies have highlighted the relevance of circRNAs in adipogenesis, but the precise molecular mechanism has largely remained elusive.

In humans, has_circ_0001982 (circRNA-RNF111) derived from the second exon of the ring finger protein 111 gene *(RNF111)* is reportedly involved in various cancers. In this study, we first confirm the existence of the circRNF111 (899 bp) in the bovine species, which is highly homologous to has_circ_0001982. We explore the endogenous functions of circRNF111 in adipogenesis and fat deposition of bovine adipocytes. Our research also confirms the targeting relationship between circRNF111 and miR-27a-3p. We prove that circRNF111 sponges miR-27a-3p to affect the expression level of *PPARγ* and eventually promotes fat deposition.

## Materials and Methods

### Tissue and cell lines

All experimental animals were dealt with as per standard procedures formulated by Chinese Council of Animals Care [GB/T 35892–2018]. The procedures were further supervised by the Experimental Animal Management Committee of the Northwest Agricultural and Forestry University. All the tissue samples from Qinchuan cattle (Bos taurus Qinchuanensis) of two development states (neonatal 3 days and adult 24 months) were collected from a livestock farm in Xi’an, P.R. China. Primary preadipocytes were isolated from the subcutaneous fat of three newborn calves, as previously described [30,31]. HEK-293 T cells were purchased from the American Type Culture Collection (ATCC). Preadipocytes were cultured in Dulbecco’s modified Eagle’s medium: Nutrient Mixture F12 (DMEM/F12, HyClone, USA) supplemented with 10% foetal bovine serum (FBS, Gibco, USA) and 1% penicillin-streptomycin solution. HEK-293 T cells were cultured in DMEM with 10% FBS. They were all cultured at 37°C with 5% CO_2_.

### Differentiation of preadipocytes

Adipocyte differentiation was induced by M1 medium [DMEM/F12 containing 10% FBS, 1% penicillin-streptomycin solution, 0.5 mM 3-isobutyl-1-methylxanthine (Sigma, USA), 1 µM dexamethasone (Sigma, USA), and 1.5 µg/mL insulin (Sigma, USA)]. Two days later, the M1 medium was replaced with M2 medium (DMEM/F12 containing 10% FBS, 1% penicillin-streptomycin solution, and 1.5 µg/mL insulin). Then, differentiation was induced for 8 days, during which the medium was changed once every 2 days. Adipocytes were collected on the 8^th^ day of adipogenic differentiation for marker gene or staining index determination.

### RNA extraction and real-time qPCR

Total RNA was extracted from cells and tissues using TRIzol reagent (Invitrogen, Carlsbad, CA, USA). The gDNA was extracted using a genomic DNA isolation kit (Sangon Biotech, Shanghai, China). The nuclear and cytoplasmic fractions were extracted using a PARIS kit (Ambion, Life Technologies). RNA was reverse transcribed with a PrimeScript™ RT reagent kit (Takara, Tokyo, Japan). Based on the sequence of circRNAs, the divergent primers were designed to determine their authenticity. miRNAs-specific stem-loop primers were used to perform reverse transcription. Real-time quantitative PCR (qPCR) reactions were performed on a Bio-Rad CFX96 Real-Time Detection System using the SYBR Green PCR Master Mix (Takara, Tokyo, Japan). Data analyses were performed using the 2 ^−ΔΔCT^ method, as described previously [32]. Cattle *GAPDH* and *U6* were used as internal controls.

### RNase R treatment and actinomycin D assay

For RNase R treatment, 2 µg of extracted RNA was incubated for 15 min at 37°C with or without 5 U/µg RNase R (Epicentre, Madison, WI, USA), and then purified with the RNeasy MinElute Cleaning kit (Qiagen, Germany). Primary adipocytes were exposed to 2 µg/mL actinomycin D (MilliporeSigma, Burlington, MA, USA) at the indicated time point. Total RNA was then extracted to test the half-life of circRNF111 and linear mRNA.

### Vector construction and cell transfection

For construction of the circRNA overexpression vector, the full-length sequence of circRNF111 was amplified to construct the pCD2.1 vector (Geneseed, Guangzhou, China). The wild-type or the mutant full-length sequence of circRNF111 was inserted into the *Xhol I-Not I* restriction sites of the psiCHECK-2 vector (Promega, Fitchburg, WI, USA). An miR-27a-3p sensor (psiCHECK-2-miR-27a-3p 3×) was created by inserting three consecutive miR-27a-3p complementary sequences into the psiCHECK-2 vector. The wild-type or mutated 3’UTR fragment of *PPARγ* containing miR-27a-3p targeted site was cloned into the psiCHECK-2 Vector at the 3’-end of the *Renilla* gene. Small interfering RNA (siRNA) oligonucleotides were designed to combine with the back-splice region. The miR-27a-3p mimics, inhibitors, and corresponding negative control (NC) were synthesized using RiboBio (Guangzhou, China). The mimics and inhibitors (50 nM), siRNA (50 nM), or vectors (2 µg/mL) were transfected into cells with Lipofectamine 2000 (Invitrogen).

### RNA FISH

CircRNF111 probes (RiboBio, Guangzhou, China) targeting the back-splicing junction region were designed to visualize circRNF111 fluorescence *in situ*. The differentiated preadipocytes were first fixed with in situ hybridization fixative. After prehybridization, cells were incubated with the labelled circRNF111 probes in hybridization buffer at 55°C overnight. Cell nuclei were counterstained by 4’,6-diamidino-2-phenylindole (DAPI; Sigma, USA). Laser confocal microscopy was used to observe the localization of circRNF111 (Nikon, Tokyo, Japan).

### Dual Luciferase Reporter Assay

HEK-293 T cells were plated in 96-well plates for 24 h before transfection. The cells were co-transfected with psi-CHECK-2 reporter plasmid, miR-27a-3p mimics, si-RNA or pCD2.1-circRNF111 vector. Cells were harvested 24 h after transfection. The ratio of Renilla and Firefly luciferase activity was detected with the Dual-Luciferase Reporter Assay Kit (#E2920, Promega, Fitchburg, WI, USA). The optical density of the resulting solution was assessed using the automatic microplate reader (Molecular Devices, Sunnyvale, CA, USA).

### RNA-binding protein immunoprecipitation (RIP)

The Magna RIP RNA-binding Protein Immunoprecipitation Kit (Millipore, Bedford, MA, USA) was adopted to confirm the relationship between circRNF111 and miR-27a-3p. Cells were briefly lysed in RIP buffer and incubated with magnetic beads, which were conjugated with anti-Argonaute2 (anti-Ago2; Abcam) or anti-immunoglobulin G (anti-IgG; Abcam). Next, proteinase K (Solarbio) was added to digest the protein, and the RNA in the immunoprecipitated product was extracted. Finally, the co-precipitated circRNF111 and miR-27a-3p was detected by real-time qPCR after reverse transcription.

## circRNF111 pull-down

Biotin-labelled circRNF111 probe and negative control probe (NC oligo probe) were synthesized by RiboBio (Guangzhou, China). We purchased the Pierce™ magnetic RNA-protein pull-down kit (#20164, Thermo, USA) and performed the experiment according to the manufacturer’s instructions. In brief, the biotin-labelled probe was bound to streptavidin magnetic beads for 30 minutes at room temperature. Then, the probe-magnetic beads complexes were incubated with the cell lysates from preadipocytes for eight hours. On the next day, the RNA in the immunoprecipitates were extracted and eluted with lysis buffer. Finally, real-time qPCR was used to detect the expression levels of circRNF111 and miRNAs in the immunoprecipitates.

### Western blot analysis

Proteins from cultured bovine preadipocytes were prepared with RIPA buffer (Solarbio, Beijing, China). Proteins were loaded onto 12% sodium dodecyl sulphate-polyacrylamide gel electrophoresis (SDS-PAGE) and transferred onto polyvinylidene difluoride (PVDF) membranes (Thermo Fisher Scientific). The membranes were incubated overnight with primary antibodies specific for anti-GAPDH (1:1,000, #ab9485, Abcam, Cambridge, UK), anti-PPARγ (1:500, #WL01800, Wanlei Bio, Shenyang, China), anti-fatty acid-binding protein (FABP4, 1:500, #bsm-51,247 M, Bioss, Beijing, China) and anti-C/EBPα (1:500, #WL01899, Wanlei Bio, Shenyang, China) at 4°C. The goat anti-mouse IgG (H&L)-horseradish peroxidase (HRP, 1:5,000, #bs-40,296 G, Bioss, China), and goat anti-rabbit IgG (H&L)-HRP (1:5,000, #bs-40,295 G, Bioss, China) were used as secondary antibodies. After incubation with secondary antibodies, the membranes were quantified with the ChemiDoc XRS system (Bio Rad, Hercules, CA, USA).

### Oil Red O and BODIPY staining

After 8 days of differentiation, the preadipocytes were stained with Oil Red O (#O0625, Sigma, USA) and 4,4-difluoro-1,3,5,7,8-pentamethyl-4-bora-3a,4a-diaza-s-indacene (BODIPY 493/503; D3922, Thermo Fisher Scientific). Oil Red O staining was performed according to the manufacturer’s instructions. To quantify the staining of fat droplets, 100% isopropanol was used to dissolve the lipid droplets, and the absorbance was measured at 510 nm. For BODIPY staining, the cells were washed with PBS for three times and fixed with 4% paraformaldehyde for 10 minutes. Hank’s Balanced Salt Solution containing 10 µM BODIPY was added to the cells and then incubated at 37°C for 30 min in the dark. The samples were washed three times with PBS and photographed immediately.

### Statistical analyses

Data are represented as the mean ± standard error (SEM) of at least three independent experiments. Statistical analyses were performed using SPSS 22.0 statistical software (SPSS, Chicago, IL, USA). Comparison of two groups was determined by Student’s t test and multiple groups by one-way ANOVA with Tukey’s post hoc test. A probability of 0.05 or less was considered statistically significant.

## Results

### Characteristics of circRNF111 in adipose tissue

**Figure 1.**
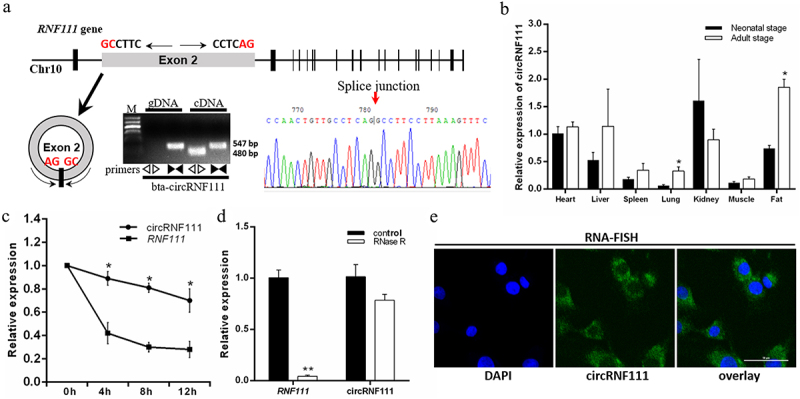
CircRNF111 identification and expression pattern in bovine adipocytes. (a, top) The genomic locus of circRNF111. (a, bottom) PCR analysis of divergent and convergent primers in cDNA and genomic DNA (gDNA). The back-splice junction (arrow) of circRNF111 was identified by Sanger sequencing. (b) The expression of circRNF111 in different tissues of cattle at two developmental stages. (c) Real-time qPCR for the abundance of circRNF111 and *RNF111* in preadipocytes treated with actinomycin D. (d) Real-time qPCR analysis of circRNF111 and *RNF111* levels with and without RNase R treatment. (e) FISH detection of circRNF111 in adipocytes. Scale bars, 50 µm. Data are presented as means ± SEM of three independent experiments. *P < 0.05. **P < 0.01.

In this study, we analysed the authenticity and stability of circRNF111 in bovine adipocytes. The results of PCR amplification and sequencing showed that circRNF111, which is highly homologous to hsa_circ_0001982, exists in bovine adipocytes. CircRNF111 is formed by circularization of the second exon sequence of the *RNF111* gene (Figure 1a). Real-time qPCR assays revealed that circRNF111 was expressed in all seven bovine tissues we selected. Moreover, the expression of circRNF111 in adipose tissue of adult cattle was significantly higher than in newborn calves (Figure 1b). Compared to linear RNA, circRNF111 was more resistant to actinomycin D treatment in preadipocytes (Figure 1c). When RNase R was used to digest the extracted RNA, circRNF111 had higher stability (Figure 1d). The results reveal that circRNF111 was mainly expressed in the cytoplasm of preadipocytes (Figure 1e).

## circRNF111 promotes preadipocytes differentiation

**Figure 2.**
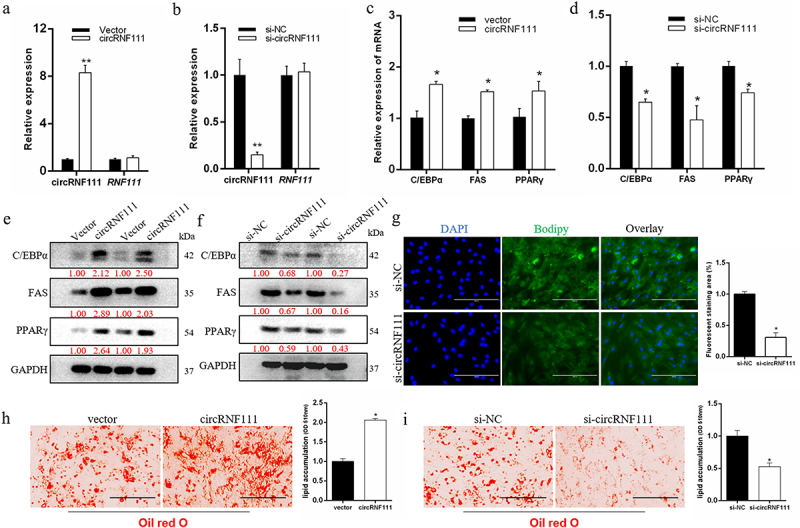
CircRNF111 promotes adipogenesis of preadipocytes. (a, b) Real-time qPCR detected the overexpression and interference efficiency of circRNF111. (c, d) The expression levels of adipogenesis marker genes were detected by real-time qPCR. (e, f) The expression levels of adipogenesis marker genes were detected by western blots. (g) Interference with si-circRNF111 in pre-adipocytes, followed by BODIPY staining to analyse lipid droplet deposition. The fluorescence signal was analysed by ImageJ software. Scale bars, 100 µm. (h, i) Lipid droplets in preadipocytes were stained with Oil Red O. Lipid contents were measured by spectrophotometric analysis after dissolution in isopropanol. Scale bars, 100 µm. Data are presented as means ± SEM of three independent experiments. *P < 0.05. **P < 0.01.

To examine the effects of circRNF111 on the biological functions of preadipocytes, the overexpression vector and siRNA of circRNF111 were successfully transfected into bovine primary preadipocytes. As illustrated in Figures 2A and 2b, overexpression or interference with circRNF111 did not cause the expression of the corresponding maternal gene *RNF111* in preadipocytes, which ensures the reliability of the follow-up research results. The results of real-time qPCR and western blots indicate that overexpression of circRNF111 significantly increases the expression of adipogenesis marker genes, including *C/EBPα, FAS*, and *PPARγ* (Figures 2C and 2e). However, interference with circRNF111 in preadipocytes inhibits the expression of these genes (Figures 2D and 2f). Since the pCD2.1 overexpression vector carries a green GFP fluorescence, we could only analyse BODIPY staining after circRNF111 interference. Eight days after adipogenic induction, the cells were subjected to BODIPY staining. The results showed that interference with circRNF111 significantly inhibits the intensity of green fluorescence in adipocytes (Figure 2g). In addition, Oil Red O staining results showed that circRNF111 significantly promotes the lipogenesis of preadipocytes (Figure 2h), whereas the accumulation of lipid droplets decreases after interference with circRNF111 (Figure 2i).

### CircRNF111 serves as a sponge for multiple miRNAs

**Figure 3.**
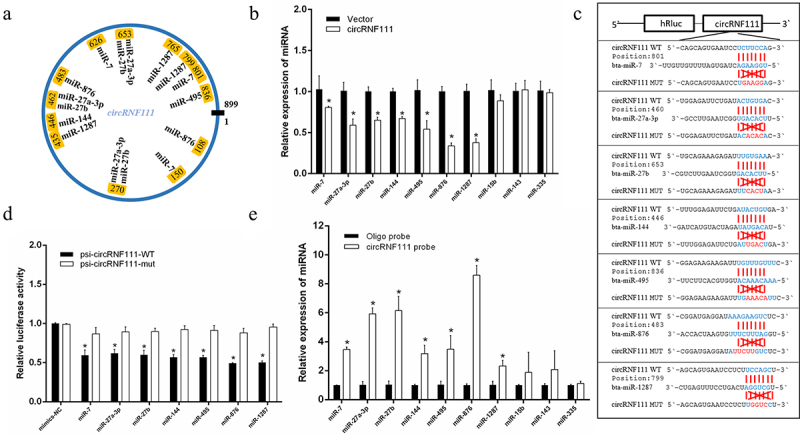
CircRNF111 serves as a sponge for multiple miRNAs. (a) Schematic drawing illustrating the putative binding sites of the miRNAs associated with circRNF111. (b) Real-time qPCR was used to detect changes in the expression of some miRNAs after overexpression of circRNF111 in bovine precursor adipocytes. (c) Sequence analysis of the binding site of circRNF111 and miRNAs. (d) Luciferase reporter assay for the luciferase activity of psi-circRNF111 or psi-circRNF111-mutant in HEK-293 T cells co-transfected with miRNA mimics. (e) CircRNA pull-down assays were performed using a specific biotin-labelled circRNF111 probe in adipocytes. Real-time qPCR was used to detect the expression levels of miRNAs in immunoprecipitates. Compared to negative control (NC) probe. Data are presented as means ± SEM of three independent experiments. *P < 0.05.

To better investigate circRNF111, we analysed the existing research on has-circRNF111. The statistical results showed that circRNF111 regulates the process of cancer by adsorbing multiple miRNAs, such as miR-27b-3p, miR-140-5P, miR-143-3p, and miR-876-3p (Supplementary Table 1) [33–40]. Because there are mutation sites in the circRNF111 sequence of cattle compared with has-circRNF111 (Supplementary Figure 1), these mutation sites may lead to changes in targeting bta-circRNF111 to the corresponding miRNAs. Our findings demonstrated that bta-circRNF111 can adsorb miR-7, miR-27a-3p, miR-27b, miR-144, miR-495, miR-876, and miR-1287 (Figure 3a). Our results indicate that unlike hsa-circRNF111, circRNF111 does not adsorb miR-143 (Supplementary Figure 2). The real-time qPCR results illustrated that overexpression of circRNF111 in bovine precursor adipocytes reduces the expression levels of these miRNAs (Figure 3b). We constructed wild-type and mutant dual-luciferase reporter vectors containing circRNF111 targeting binding sites (Figure 3c). The results showed that the expression of the Renilla luciferase gene is inhibited when the wild-type vector is co-transfected with miRNA mimics. The luciferase activity of the psi-circRNF111-WT +miRNAs group was significantly lower than that of the control group (Figure 3d). A biotin-labelled circRNF111 probe was used to conduct RNA pull-down assays to validate this adsorption relationship. After the extraction of RNA from the immunoprecipitation complex, real-time qPCR detected a high expression of miRNAs (Figure 3e).

### CircRNF111 serves as a sponge for miR-27a-3p

**Figure 4.**
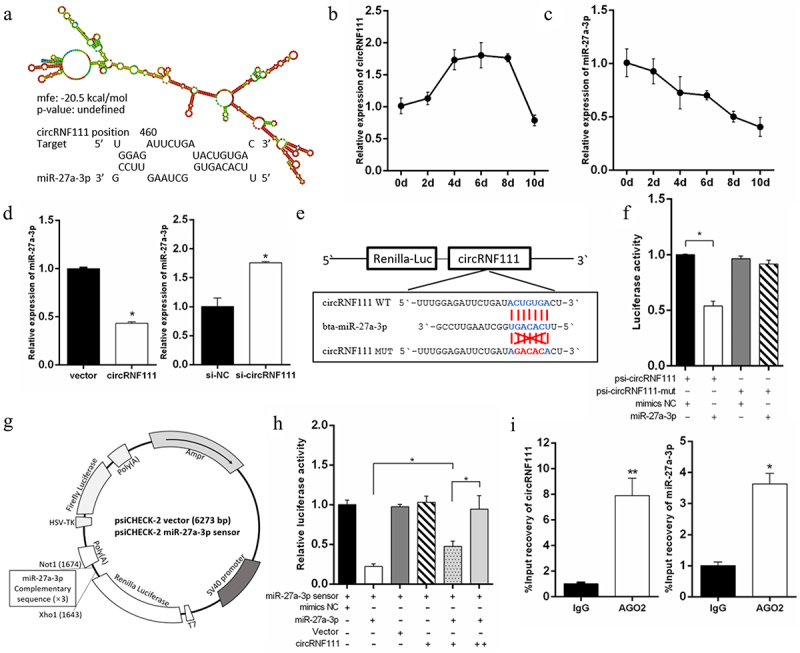
CircRNF111 serves as a miR-27a-3p sponge. (a) Predicted circRNF111 secondary structure and binding site to miR-27a-3p. (b, c) Expression dynamics of circRNF111 and miR-27a-3p during adipocytes differentiation. (d) After overexpression and interference with circRNF111, real-time qPCR was used to detect the expression of miR-27a-3p. (e) Sequence analysis of the binding site of circRNF111 and miR-27a-3p. (f) Luciferase reporter activity of circRNF111-WT and circRNF111-mut in HEK-293 T cells co-transfected with miR-27a-3p mimics or NC. (g) Schematic diagram of psi-CHECK2 vector for miR-27a-3p sensor (psiCHECK2-miR-27a-3p 3×). (h) The miR-27a-3p sensor was transfected into HEK-293 T cells, together with mimics-NC, miR-27a-3p mimics, pCD-2.1-non, or pCD2.1-circRNF111. Luciferase activities were measured after transfection. (i) Ago2-RIP assay for the amount of circRNF111 and miR-27a-3p. Data are presented as means ± SEM of three independent experiments. *P < 0.05. **P < 0.01.

To investigate the potential mechanism of circRNF111 in regulating adipogenesis, starBase v2.0 and RNA-hybrid software were used to predict the potential target miRNA of circRNF111. The data showed that miR-27a-3p contained the potential complementary sequences of circ-RNF111 (Figure 4a). In addition, the expression of circRNF111 increased during the differentiation of preadipocytes and decreased after 8 days of culture (Figure 4b). However, the expression of miR-27a-3p decreased with the process of differentiation (Figure 4c). The results of real-time qPCR indicated that overexpression of circRNF111 in preadipocytes leads to a decrease in the detected expression of miR-27a-3p, while interference with circRNF111 increases the expression of miR-27a-3p (Figure 4d). Dual-luciferase reporter assay showed that the co-transfection of psiCHECK2-circRNF111 wild type and miR-27a-3p mimics decreases the luciferase activity compared to the control group (Figures 4E and 4f). To verify the binding relationship between circRNF111 and miR-27a-3p, we first generated a miR-27a-3p sensor by inserting three copies of complementary fragments into the psiCHECK-2 vector, as illustrated in Figure 4g. The results showed that miR-27a-3p overexpression inhibits the Renilla luciferase expression of the biosensor vector. However, co-transfection of circRNF111 alleviated the inhibitory effect of miR-27a-3p and had a dose effect (Figure 4h). To further verify this targeting relationship, we used AGO2 protein antibody to perform immunoprecipitation analysis. The results indicated that both circRNF111 and miR-27a-3p are highly expressed in the immunoprecipitates (Figure 4i).

### Interfering with miR-27a-3p promotes adipogenesis of preadipocytes

**Figure 5.**
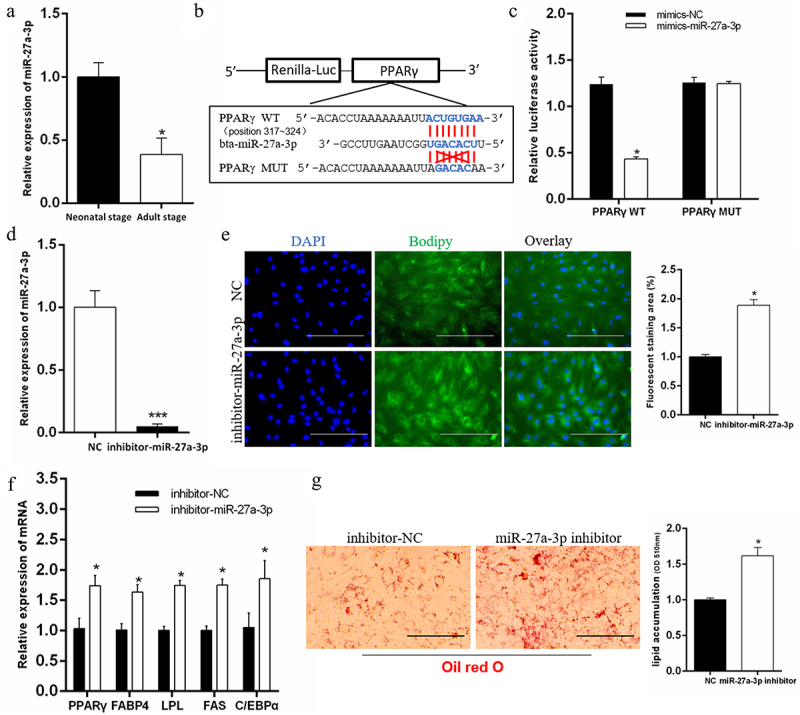
Interfering with miR-27a-3p promotes adipogenesis of preadipocytes. (a) The expression difference of miR-27a-3p in adipose tissue of newborn calves and adult calves. (b, c) The dual fluorescence reporter system verified the targeting relationship between miR-27a-3p and *PPARγ* gene. (d) Real-time qPCR was used to detect the interference efficiency of miR-27a-3p. (e) Interference with miR-27a-3p in preadipocytes, followed by BODIPY staining to analyse lipid droplet deposition. Scale bars, 100 µm. (f) The mRNA levels of adipogenesis-related genes in bovine preadipocytes with miR-27a-3p inhibition. (g) Lipid droplets in preadipocytes were stained with Oil Red O after interference with miR-27a-3p. Lipid contents were measured by spectrophotometric analysis after dissolution in isopropanol. Scale bars, 100 µm. Data are presented as means ± SEM of three independent experiments. *P < 0.05. ***P < 0.001.

To determine that circRNF111 promoted adipogenesis by adsorbing miR-27a-3p, we first analysed the physiological function of miR-27a-3p in preadipocytes. We found that the expression of miR-27a-3p in adult calf fat is significantly lower than that of newborn calves, which indicates that miR-27a-3p may have a negative regulatory effect on adipogenesis (Figure 5a). Through the prediction of TargetScan 7.0 and the verification of a dual fluorescence reporter system, we discovered that *PPARγ* is the target gene of miR-27a-3p (Figures 5B and 5c). Furthermore, we designed miR-27a-3p inhibitors to transfect preadipocytes (Figure 5d). After eight days of cell differentiation, BODIPY staining results showed that miR-27a-3p interference significantly promotes adipogenesis (Figure 5e). In addition, the real-time qPCR results showed that the interference of miR-27a-3p increases the expression of adipogenesis-related genes (figure 5f). Oil Red O staining results showed that the accumulation of lipid droplets increases after interference with miR-27a-3p (Figure 5g).

## circRNF111 regulates preadipocytes adipogenesis through miR-27a-3p

**Figure 6.**
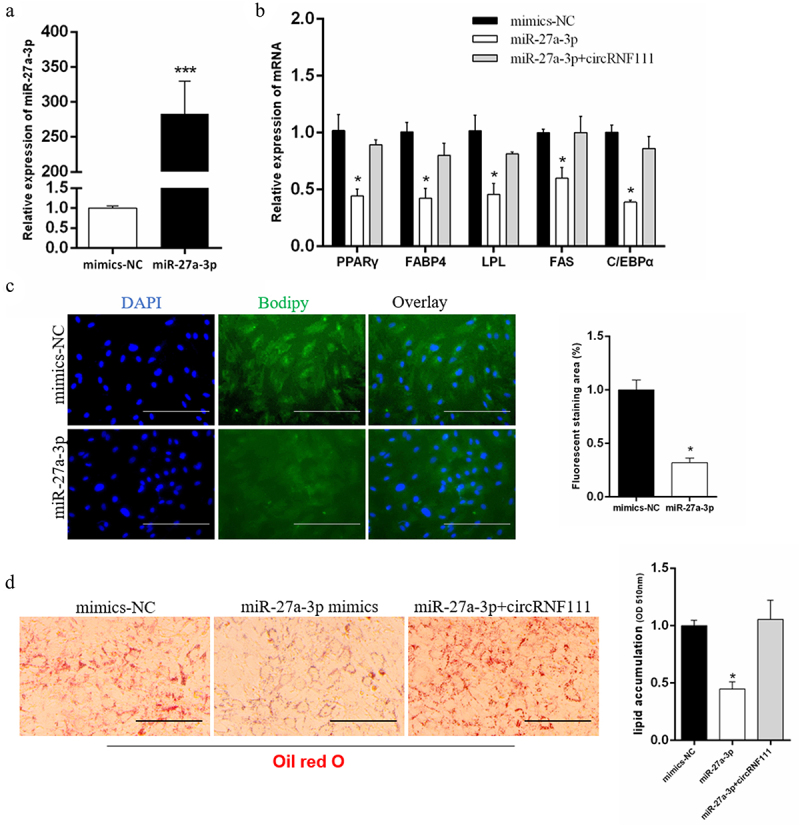
CircRNF111 regulates preadipocytes adipogenesis through miR-27a-3p. (a) Real-time qPCR was used to detect the overexpression efficiency of miR-27a-3p. (b) The mRNA levels of adipogenesis related genes in preadipocytes with miR-27a-3p mimics alone or co-transfected with circRNF111. (c) Overexpression with miR-27a-3p in preadipocytes, followed by BODIPY staining to analyse lipid droplet deposition. Scale bars, 100 µm. (d) Lipid droplets in preadipocytes were stained with Oil Red O. Lipid contents were measured by spectrophotometric analysis after dissolution in isopropanol. Scale bars, 100 µm. Data are presented as means ± SEM of three independent experiments. *P < 0.05. ***P < 0.001.

To further determine the mechanism of circRNF111, we co-transfected miR-27a-3p mimics (Figure 6a) with circRNF111 in preadipocytes. We found that transfected miR-27a-3p alone can inhibit the expression of adipogenesis-related genes, and the expression of these genes increases after co-transfection with circRNF111 (Figure 6b). Since the circRNF111 overexpression vector has its own green fluorescence, we did not perform BODIPY staining after co-transfection miR-27a-3p mimics with circRNF111. The results of BODIPY and Oil Red O staining showed that overexpression of miR-27a-3p inhibits lipid droplet formation (Figures 6C and 6d). The lipid droplet formation was restored after co-transfection of miR-27a-3p mimics and circRNF111 vector (Figure 6d).

### *CircRNF111 facilitates adipogenesis by relieving repression of miR-27a-3p for* PPARγ *expression.*

**Figure 7.**
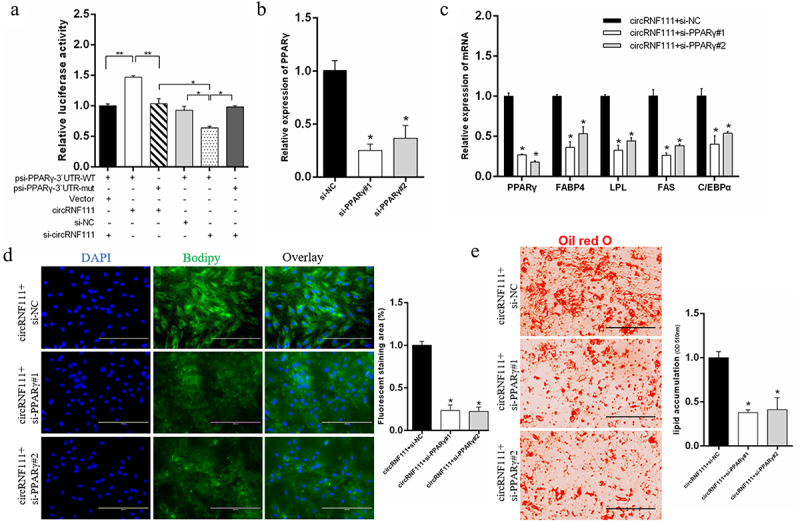
CircRNF111 promotes preadipocytes adipogenesis in a miR-27a-3p/*PPARγ* dependent manner. (a) Luciferase reporter activity of *PPARγ* 3`UTR in HEK293T cells with circRNF111 knockdown or overexpression. Data are presented as means ± SEM of six independent experiments. (b) Real-time qPCR was used to detect the overexpression and interference efficiency of si-*PPARγ*. (c) The mRNA levels of adipogenesis-related genes in preadipocytes with circRNF111 alone or co-transfected with si-*PPARγ*. (d) After transfection with circRNF111 alone or co-transfection with si-*PPARγ*, BODIPY staining was used to analyse the lipid droplet content. Scale bars, 100 µm. (e) Lipid droplets were stained with Oil Red O with circRNF111 alone or co-transfection with si-*PPARγ*. Scale bars, 100 µm. Data are presented as means ± SEM of three independent experiments. *P < 0.05. **P < 0.01.

Due to the targeting relationship between *PPARγ* and miR-27a-3p, we wanted to explore whether circRNF111 increases adipogenesis in a *PPARγ*-dependent manner. We found that circRNF111 overexpression or knockdown can further increase or reduce the luciferase activity of the *PPARγ* wild-type reporter (Figure 7a). This result indicates that circRNF111 affects the expression of the psiCHECK2-*PPARγ* vector by adsorbing the existing miR-27a-3p in cells. In this study, the si-*PPARγ* significantly inhibited the expression of the *PPARγ* gene (Figure 7b). Real-time qPCR results showed that, compared with the circRNF111 overexpression group, co-transfection of circRNF111 and si-*PPARγ* can significantly inhibit the expression of adipogenesis-related genes (Figure 7c). The results of BODIPY (Figure 7d) and Oil Red O staining (Figure 7e) showed that, compared with the circRNF111 overexpression group, co-transfection of circRNF111 and si-*PPARγ* can significantly inhibit the formation of lipid droplets and inhibit fat differentiation.

## Discussion

For humans, obesity has become a major health threat. Fat development and metabolism disorders directly lead to human diseases [41]. For livestock, fat development is closely related to meat quality and feed conversion rate [42,43]. Multiple methods have been used to improve the palatability and acceptability of meat for the consumer, primarily by increasing the amount of marbling [1]. To circumvent the obvious waste of resources and resultant inefficiency in production incurred by the overfattening of livestock, scientists have attempted to understand the regulation of adipose tissue differentiation. The data consistently indicate that intramuscular and subcutaneous adipose tissues are metabolically distinct. Therefore, it is critical to investigate adipocyte biology and regulation in food-production animals.

Fat formation is an inclusive term describing the proliferation, differentiation, and conversion of cells into lipid-assimilating cells [44,45]. Adipocytes are derived from embryonic mesenchymal stem cells (eMSCs). Cells within budding embryonic and foetal adipose tissue depots undergo adipogenesis in a stagnant manner, and the formation of postnatal adipocytes is more rapid [46]. Numerous studies have stated that, in addition to coding genes, non-coding RNAs are involved in the regulation of lipid deposition [3,5,18,47,48]. Therefore, we paid attention to the role of circRNF111 in adipogenesis. Like all known circRNAs, the circRNF111 has more stable properties than linear RNA under conditions of RNase R and actinomycin D treatment. In addition, the FISH results showed that the high distribution of circRNF111 in the cytoplasm also guarantees its adsorption of miRNAs. In addition, circRNF111 is highly homologous to human has_circ_0001982, and there are many human cancer-related studies. Tang et al. declared that has_circ_0001982 functioned as an oncogene in breast cancer through decreasing miR-143 [35]. In addition, has_circ_0001982 is involved in the regulation of growth, migration, and invasion of gastric cancer cells by binding to miR‑27b‑3p [33].

However, Lin et al. suggested that has-circRNF111 inhibition enhanced insulin resistance and lipid deposition through regulating the miR-143-3p-*IGF2R* cascade [36]. In our study, we confirmed that circRNF111 is markedly overexpressed in adult adipose tissue. We speculate that circRNF111 functions in bovine adipocytes in complete contrast to human adipocytes. Based on these speculations, we used overexpression and interference to explore whether circRNF111 has important functions in bovine fat development. The functional gain and loss experiments demonstrated that circRNF111 was associated with the cell adipogenesis. After overexpression of circRNF111 in adipocytes by circRNA-specific vectors, we detected a significant increase in the expression of FAS, which is the key gene in adipogenic process. In addition, adipogenesis pathway-related genes were also significantly overexpressed. Combined with molecular biology techniques, we found that circRNF111 can significantly initiate the differentiation of preadipocytes and promote the formation of lipid droplets (Figure 2).

To explain this functional difference, we analysed the targeting of bta-circRNF111 with miR-143. We found that bovine circRNF111 has several base mutations compared with hsa-circRNF111. As a result, these miR-143 binding sites cannot bind again (Supplementary Figure 2). This mutation eventually changes the downstream function from the level of adsorbed miRNA. Our results demonstrate functional differences resulting from circRNAs sequence mutations. This also reminds us that in-depth analytical mechanisms should be performed in circRNAs functional studies. Moreover, combining with bioinformatics, dual-luciferase reporter assays, and anti-Ago2 RNA immunoprecipitation, we found that circRNF111 could also adsorb more miRNAs, such as miR-27a-3p, miR-144, miR-876, and miR-1287. These results suggest that circRNF111 may have more abundant functions on cattle.

To investigate the function of circRNF111 in fat deposition, we focused on miR-27a-3p because it has been reported that miR-27a-3p could inhibit the differentiation of adipocytes [47]. In our study, overexpression of miR-27a-3p inhibited the expression of adipogenesis marker genes and the formation of lipid droplets. Existing reports indicate that circRNAs function mainly as a miRNA molecular sponge and contain numbers and types of miRNA response elements at varying degrees [49]. The same results were obtained in our study. Overexpression and interference with circRNF111 in preadipocytes significantly affected intracellular miR-27a-3p expression. After the co-transfection of the psi-CHECK2-circRNF111^WT^ vector and miR-27a-3p mimics, the circINSR sequence, which contained in the 3’-UTR region of Renilla luciferase, was recognized by miR-27a-3p, decreasing the *Renilla: Firefly* ratio in the final system, which indirectly supports a targeting relationship between circRNF111 and miR-27a-3p. Furthermore, the binding sites of circRNF111 for miR-27a-3p were validated by RNA pull-down and RNA binding protein immunoprecipitation (RIP analyses).

This study also verified the targeting relationship between miR-27a-3p and its downstream target gene *PPARγ. PPARγ* was proved to be the most important regulatory factor for adipocytes to perform biological functions [50]. To further verify whether circRNF111 could function by miR-27a-3p, we co-transfected circRNF111 and si-*PPARγ* in bovine primary myocytes. We found that overexpression of circRNF111 and then interference with *PPARγ* may reverse the role of circRNF111 in the adipogenic pathway. Results of the co-transfection experiments also showed that circRNF111 could abolish the endogenous suppressive effect of miR-27a-3p on the target gene *PPARγ*.

In summary, we found that co-transfection of circRNF111 and miR-27a-3p partially offset the inhibitory effect of miR-27a-3p on adipogenesis. CircRNF111 regulated the expression of *PPARγ* by adsorbing miR-27a-3p. Transfection of si-*PPARγ* after circRNF111 overexpression can cancel the differentiation-promoting effect of circRNF111. Our study highlights the positive effect of circRNF111 on adipogenesis, and circRNF111 promotes preadipocytes differentiation in a miR-27a-3p/*PPARγ* dependent manner. However, it is still unknown whether circRNF111 can regulate other miRNAs. Future research should be done to interrogate the in-depth regulation mechanisms of circRNF111.
